# Efficient Mineralization of Fluoroelastomers Using Superheated Water in the Presence of Potassium Hydroxide

**DOI:** 10.3390/molecules28207057

**Published:** 2023-10-12

**Authors:** Jin Hamaura, Hisao Hori, Ayane Fujishima, Hirofumi Mukae

**Affiliations:** 1 Faculty of Science, Kanagawa University, 3-27-1 Rokkakubashi, Kanagawa-ku, Yokohama 221-8686, Japan; 2 Technology Innovation Center, Daikin Industries, Ltd., 1-1 Nishi-Hitotsuya, Settsu 566-8585, Japan

**Keywords:** decomposition, fluoroelastomer, fluoropolymer, mineralization, subcritical water, superheated water

## Abstract

The mineralization of fluoroelastomers (FKMs) in superheated water in the presence of potassium hydroxide (KOH) was investigated with the aim of developing a methodology for recycling the fluorine element. Two FKMs—an “uncrosslinked FKM”, representing a poly(vinylidene fluoride-*co*-hexafluoropropylene) (poly(VDF-*co*-HFP)) copolymer with a VDF/HFP molar ratio of 78/22 and a “crosslinked FKM” consisting of this copolymer (cured by peroxide) and carbon black—were treated. The fluorine content of these FKMs was efficiently transformed into F^−^ ions in the reaction solution using low KOH concentrations (0.10–0.50 M) at 200–250 °C. When the uncrosslinked or crosslinked FKMs reacted with aqueous KOH (0.20 M) at a rather low temperature (200 °C) for 18 h, the fluorine content of these FKMs was completely mineralized (both F^−^ yields were 100%). Although the crosslinked FKM contained carbon black, the fluorine mineralization of the FKM was not inhibited. The addition of Ca(OH)_2_ to the reaction solutions after the superheated water treatment at 250 °C for 6 h with aqueous KOH (0.50 M) led to the production of pure CaF_2_, identified using X-ray spectroscopy, with 100% and 93% yields for the uncrosslinked and crosslinked FKMs, respectively.

## 1. Introduction

Fluoropolymers are functional materials that are used in numerous kinds of equipment because they have a high thermal and chemical resistibility [1,2,3,4,5,6]. Among the various types, fluoroelastomers (FKMs), which consist of vinylidene fluoride (VDF) and other monomers, are functional rubber materials that have a low gas permeability and high resistance to heat, oil, organic solvents, acids, ozone, steam, and flames. Thus, they have a variety of usages, such as applications in electric wiring, fuel tubes, O-rings, gaskets, and valve seats [7,8,9,10]. 

As fluoropolymer production has increased, waste treatment technologies must meet this increasing demand [11]. Incineration is one of the suitable measures for these polymer wastes [12]. However, the generated gaseous hydrogen fluoride (HF) significantly shortens the life of the refractory bricks of incinerators. Some wastes of polytetrafluoroethylene (PTFE), which is the most widely manufactured fluoropolymer, are reused in ram extrusion products after purification or changed to PTFE with a lower mass through heat addition (typically using electron beams) and used again as a fine powder combined with other ingredients [4]. However, even with such reuses, the recycling rate of fluoropolymers is estimated to be only 3.4% in Europe [13].

If fluoropolymer wastes are completely converted to fluoride ions (F^−^) (that is, subjected to mineralization), the F^−^ ions can form calcium fluoride (CaF_2_) through a reaction with calcium hydroxide (Ca(OH)_2_). The mineral of CaF_2_ is fluorite, which is the natural source of fluorine. Currently, the supply of high-purity fluorite for use in the manufacture of hydrofluoric acid is localized in two or three countries [14]. Because all fluorinated chemicals are synthesized from hydrofluoric acid, efficient mineralization technologies for fluoropolymers can help to close the loop on the fluorine element, releasing CaF_2_, representing the starting material of the fluorochemical industry [15].

Superheated water is liquid-state water at temperatures ranging between 100 °C and 374 °C (critical temperature). Reaction in superheated water is an environmentally benign waste treatment methodology because it allows for the production of useful compounds or for the conversion of waste into naturally occurring compounds [16,17,18,19,20].

We previously reported that poly(vinylidene fluoride) (PVDF) and poly(vinylidene fluoride-*co*-hexafluoropropylene) (poly(VDF-*co*-HFP)) copolymers (where the VDF/HFP molar ratio was 95/5, with this copolymer denoted as poly(VDF_95_-*co*-HFP_5_)) were efficiently mineralized in superheated water at 250 °C in the presence of an alkaline reagent [21]. Although the poly(VDF_95_-*co*-HFP_5_) copolymer can be efficiently mineralized and the poly(VDF-*co*-HFP) copolymer is a main component of FKMs, the copolymer used for FKMs has a higher HFP content, e.g., 20 mol％ [7]. Furthermore, in the market, FKMs are available not only as pure poly(VDF-*co*-HFP) copolymer rubbers but also as filler-included grades (incorporating a cure system), where the typical filler is carbon black [7]. 

The recycling of fluoroelastomers is challenging. Schuster et al. surveyed the recycling of poly(VDF-*ter-*HFP-*ter*-TFE) terpolymer (which is also a FKM) and indicated that only two methods were found to be industrially acceptable: the milling of FKMs into a fine powder which is added to virgin FKMs, and the mechanical devulcanization of FKMs which are then added to virgin FKMs [22]. In other words, no chemical recycling is involved in these methods. In the case of PTFE, gaseous TFE monomers can be regained from PTFE through pyrolysis, and a pilot plant based on this method is currently in operation [23]. However, a similar treatment cannot be applied to FKMs because VDF-based copolymers undergo dehydrofluorination through pyrolysis, which does not lead to monomer formation [24].

In this study, we investigated the reactivity of two FKMs in superheated water. The first FKM was an “uncrosslinked FKM”, i.e., a poly(VDF_78_-*co*-HFP_22_) copolymer; the second FKM was a “crosslinked FKM” that consisted of a poly(VDF_78_-*co*-HFP_22_) copolymer (cured with peroxide) and carbon black. This is the first report on the decomposition of FKMs in superheated water. The CaF_2_ formation upon the addition of Ca(OH)_2_ to the resulting aqueous phase was also achieved to close the loop on the fluorine element.

## 2. Results and Discussion

### 2.1. Decomposition of Uncrosslinked FKM

#### 2.1.1. Effect of potassium hydroxide (KOH)

The F^−^ yield, the remaining total organic carbon (TOC) ratio, and the CO_2_ yield from each FKM were calculated using Equations (1)–(3), respectively.

F^−^ yield = [(F^−^ moles in the reaction solution)/(fluorine atom moles in the initial FKM)] (1)

TOC ratio = [(TOC moles in the reaction solution)/(carbon atom moles in the initial FKM)](2)

CO_2_ yield = [(CO_2_ moles in the gas phase)/(carbon atom moles in the initial FKM)] (3)

**Figure 1:**
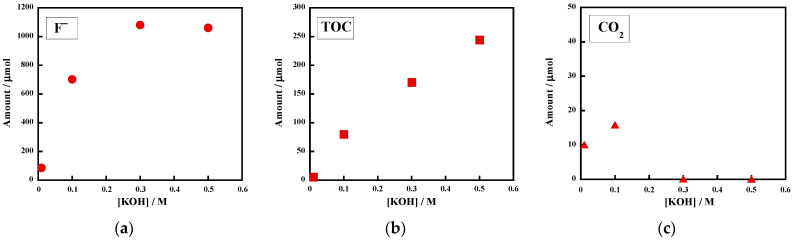
Effect of initial KOH concentration on the amounts of (**a**) F^−^ ions, (**b**) TOC, and (**c**) CO_2_. Uncrosslinked FKM (i.e., poly(VDF_78_-*co*-HFP_22_) copolymer, 30.0–30.5 mg) was reacted with aqueous KOH (0.01–0.50 M) at 250 °C for 6 h. The reaction atmosphere was argon.

Initially, the effect of the KOH concentration on the reactivity of the uncrosslinked FKM in superheated water was investigated. Figure 1 shows the KOH concentration (0.01–0.50 M) dependences of (a) the F^−^ amount in the reaction solution, (b) the TOC amount in the reaction solution, and (c) the CO_2_ amount in the gas phase generated from the reactions of the uncrosslinked FKM at 250 °C for 6 h. When the reaction was carried out without KOH, there was a trace amount of F^−^ (0.9 μmol; yield, 0%), and the TOC amount was below the limit of detection (Table 1, entry 1, not shown in Figure 1). The CO_2_ amount (1.6 μmol) was also negligible (0% yield). These results clearly indicate that the uncrosslinked FKM is stable in pure superheated water at 250 °C. We previously reported that PVDF did not decompose under the same reaction conditions (Table 1, entry 2) [21]. The uncrosslinked FKM has HFP units in addition to the VDF units that compose PVDF. Because the HFP units are perfluorinated, it is not surprising that the uncrosslinked FKM does not decompose under the conditions in which PVDF is stable.

In contrast, the reactions with KOH at 250 °C substantially enhanced the reactivity of the uncrosslinked FKM. When 0.10 M of KOH was used, the amount of F^−^ increased to 702 μmol, which corresponds to a 68% yield (Figure 1a and Table 1, entry 3). When the KOH concentration was further increased to 0.50 M, at which the molar excess was 4.8-fold relative to the amount of fluorine atoms (1036 μmol) in the initial uncrosslinked FKM (30.5 mg), the F^−^ amount increased to 1060 μmol, which corresponds to a 102% yield (Table 1, entry 5). Therefore, the fluorine content of the initial uncrosslinked FKM was completely mineralized. We previously reported that PVDF formed F^−^ ions with an 83% yield after a reaction with 0.125 M of KOH at 250 °C for 6 h (Table 1, entry 4) [21]. This value was higher than the yield (68%) for the uncrosslinked FKM under similar reaction conditions (0.10 M of KOH, Table 1, entry 3). These results suggest that the hydroxide-ion-induced decomposition of the uncrosslinked FKM preferentially proceeds at the VDF units, not at the HFP units, because the degradation is initiated from the deprotonation of the polymer chain (HFP units have no C-H bond).

The TOC amount was enhanced with the increase in the KOH concentration up to 0.50 M (Figure 1b). At 0.50 M of KOH, the amount of TOC was 244 μmol, or 31% of the carbon molar amount in the initial uncrosslinked FKM (Table 1, entry 5). A small amount of CO_2_ was detected in the gas phase, and this became a trace amount when the reactions were performed with 0.30 M or 0.50 M of KOH (Figure 1c). When 0.30 M of KOH was employed, the pH of the resulting aqueous phase was 12.7. The pH value increased to 13.1 at 0.50 M of KOH. In such basic solutions, even if CO_2_ was generated, most of the molecules were converted into CO_3_^2–^ in the resulting aqueous phase. Hence, minimal CO_2_ formation in the gas phase is reasonable. While a minimal amount of CO_2_ was formed, black solid residues appeared when the reactions were performed in the presence of KOH.

The effect of coexisting gas was also examined. When the reaction was carried out at 250 °C with 0.50 M of KOH under O_2_ instead of argon, the F^−^ yield reached 102% (Table 1, entry 6), which was the same as that under argon (F^−^ yield, 102%, Table 1, entry 5). That is, the fluorine content of the uncrosslinked FKM was completely mineralized. In contrast, the remaining TOC ratio (15%, Table 1, entry 6) was lower than that obtained under argon (31%, Table 1, entry 5). This difference suggests that O_2_ acts as an oxidizing agent to mineralize the organic component in the reaction solution.

#### 2.1.2. Mineralization at Lower Temperatures

**Figure 2:**
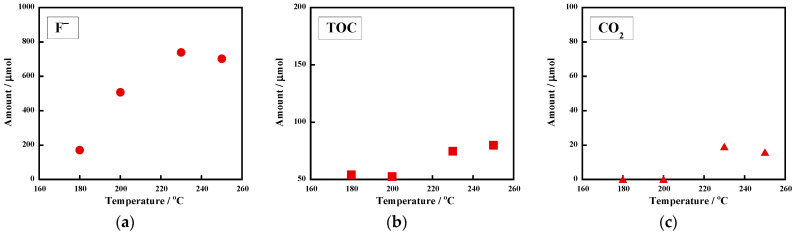
Temperature dependences of the amounts of (**a**) F^−^ ions, (**b**) TOC, and (**c**) CO_2_. Uncrosslinked FKM (30.2–30.5 mg) was treated with 0.10 M of KOH for 6 h. The reaction atmosphere was argon.

For industrial processes, it is preferable to lower both the reaction temperature and KOH concentration. Figure 2 displays the temperature dependences of the amounts of (a) F^−^ in the reaction solution, (b) TOC in the reaction solution, and (c) CO_2_ in the gas phase formed in the reactions with a rather low KOH concentration (0.10 M) for a constant reaction time of 6 h. When the reaction was carried out at 180 °C, the F^−^ amount was 170 μmol, or a 17% yield. The F^−^ amount increased with the rising temperature (Figure 2a). At 230 °C, the amount was 739 μmol (72% yield). A further increase in the temperature did not enhance F^−^ formation: at 250 °C, the F^−^ yield was 68% (Table 1, entry 3).

Although prolonging the reaction time to 18 h at 230 °C increased the F^−^ yield to 81%, complete mineralization was not obtained. In contrast, when the reaction was performed at 250 °C for 18 h, the F^−^ yield was 102%. These results indicate that 250 °C was required to induce complete fluorine mineralization through the use of 0.10 M of KOH. Figure 2b shows the temperature dependence of the TOC amount generated from the reactions using 0.10 M of KOH. While the TOC amount was negligible at 180 °C and 200 °C, it increased at higher temperatures (230 °C and 250 °C). In contrast, F^−^ ions were clearly detected even at 180 °C and 200 °C (Figure 2a). This difference suggests that defluorination occurred first, followed by chain scission, releasing organic molecules into the reaction solution. When the reaction was carried out at 230 °C or 250 °C, a trace amount (below 0.01 μmol) of 1,3,5-trifluorobenzene was found in the gas phase. This observation is consistent with the occurrence of chain scission in the VDF units.

Figure 2c displays the temperature dependence of the CO_2_ amount in the gas phase. When the reactions were performed at 180 °C and 200 °C, CO_2_ formation was at the trace level (both 0.1 μmol), where the pH values of the resulting solution were 12.6 and 12.1, respectively. The elevation of the temperature to 230 °C and 250 °C increased the CO_2_ amount to 19.1 μmol and 15.9 μmol, and under these conditions, the pH values were 8.2 and 8.4, respectively. Although the CO_2_ amount increased at higher temperatures (230 °C and 250 °C), the amount at 230 °C corresponded to only 2% of the carbon molar amount of the initial FKM. Therefore, gaseous CO_2_ was not a major product of the carbon content of the uncrosslinked FKM. To reduce the temperature in order to achieve complete fluorine mineralization, reactions with 0.20 M of KOH were performed.

When the reaction was carried out at 230 °C for 6 h, the amount of F^−^ ions reached 1041 μmol, or a 102% yield. Under these conditions, the amount of TOC was 202 μmol, or 26% of the remaining ratio, and the CO_2_ amount was 0.10 μmol, or a 0% yield. We further reduced the reaction temperature to 200 °C. When the uncrosslinked FKM was reacted for 18 h, the F^−^ amount reached 1008 μmol, or a 100% yield (Table 1, entry 7, the average value of two reactions). These are the reaction requirements for complete fluorine mineralization at the lowest temperature and KOH concentration among those tested.

#### 2.1.3. Proposed Reaction Mechanism

The hydroxide-ion-induced decomposition mechanism of the uncrosslinked FKM can be considered according to the literature [21,25]. First, OH^−^ induces the deprotonation of the methylene moiety in the VDF units (Equation (4)):

–CH_2_CF_2_–CH_2_CF_2_–CH_2_CF_2_– + OH^−^ → –CH_2_CF_2_–CH^(−)^CF_2_–CH_2_CF_2_– + H_2_O (4)

The generated unstable anion yields a C=C bond in the polymer chain, which can release F^−^ into the aqueous phase (Equation (5)):

–CH_2_CF_2_–CH^(−)^CF_2_–CH_2_CF_2_– → –CH_2_CF_2_–CH=CF–CH_2_CF_2_– + F^−^ (5)

A further deprotonation in the polymer chain generates an additional C=C bond and F^−^ (Equations (6) and (7)).

–CH_2_CF_2_–CH=CF–CH_2_CF_2_– + OH**^−^** → –CH_2_CF_2_–CH=CF–CH^(−)^CF_2_– + H_2_O (6)

–CH_2_CF_2_–CH=CF–CH^(−)^CF_2_– → –CH_2_CF_2_–CH=CF–CH=CF– + F^−^ (7)

The presence of trace 1,3,5-trifluorobenzene can be explained by the chain scission that occurs in defluorination processes. Consistently, 1,3,5-trifluorobenzene was detected as a pyrolysis product of PVDF [24] and the poly(VDF-*co*-HFP) copolymer [26].

The uncrosslinked FKM contains VDF-HFP-VDF units. As described above, hydroxide-ion-induced decomposition is likely to occur preferentially at the VDF units. However, F^−^ may also be released from the VDF-HFP-VDF units [27], as illustrated in Equations (8) and (9).

–CH_2_CF_2_–CF_2_C(CF_3_)F–CH_2_CF_2_– + OH^−^ → –CH_2_CF_2_–CF_2_C(CF_3_)F–CH^(−)^CF_2_– + H_2_O (8)

–CH_2_CF_2_–CF_2_C(CF_3_)F–CH^(−)^CF_2_– → –CH_2_CF_2_–CF_2_C(CF_3_)=CHCF_2_– + F^−^ (9)

**Figure 3:**
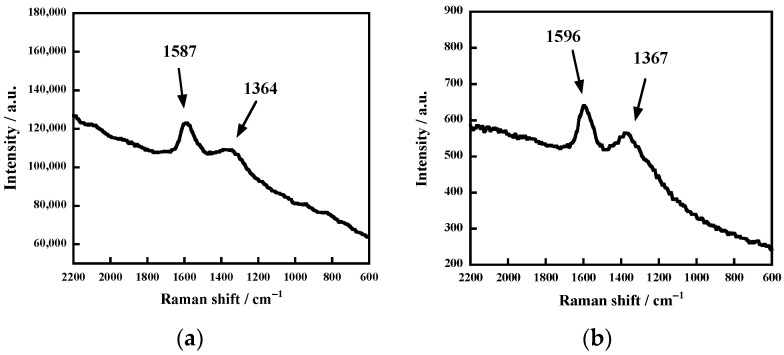
Raman spectra of the resulting residues from (**a**) uncrosslinked FKM and (**b**) PVDF. For (**a**), the uncrosslinked FKM was reacted in 0.10 M of KOH at 250 °C for 18 h. For (**b**), PVDF was heated with 1.0 M of KOH at 250 °C for 6 h [21].

The sequence of these steps generates a carbon-rich residue. Consistently, the Raman spectrum of the residue generated from the reaction of the uncrosslinked FKM under the conditions that resulted in complete fluorine mineralization showed two intense peaks at around 1587 and 1364 cm^–1^ (Figure 3a). This pattern was very similar to that of amorphous carbon [28,29,30], where the two peaks can be ascribed to the stretching of C=C bonds. The present spectrum was almost identical to that observed for the residue generated from PVDF under the conditions that resulted in complete fluorine mineralization (Figure 3b). These observations indicate that the uncrosslinked FKM generated amorphous carbon, similar to that formed from the PVDF.

### 2.2. Decomposition of Crosslinked FKM

#### 2.2.1. Effect of KOH

**Figure 4:**
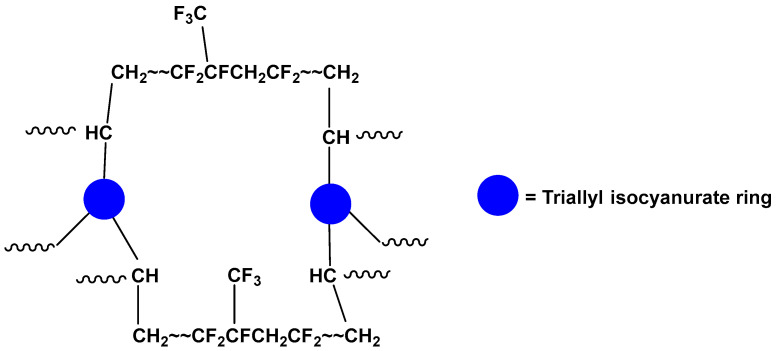
Schematic view of the structure of the crosslinked FKM prepared using a peroxide cure system. The crosslinker is triallyl isocyanurate. Filler is not shown.

Because the uncrosslinked FKM was efficiently defluorinated to release F^−^ into the reaction solution, we performed reactions of the crosslinked FKM. This sample consisted of a poly(VDF_78_-*co*-HFP_22_) copolymer and carbon black with a 100/40 weight ratio, where the polymer chain was crosslinked (Figure 4) [7,8]. When the crosslinked FKM was reacted in the absence of KOH at 250 °C for 6 h, the amount of F^−^ ions was 8.8 μmol, or a 1% yield (Table 1, entry 8). In addition, the TOC amount was below the detection limit (Table 1, entry 8). These observations indicate that the crosslinked FKM was relatively unreactive in pure superheated water at 250 °C. However, this reaction resulted in 33.1 μmol of CO_2_, which corresponds to a 3% yield (Table 1, entry 8). The pH of the resulting reaction solution was 3.5, a level which enables CO_2_ to be present in the gas phase. The origin of the CO_2_ seems not to be the poly(VDF_78_-*co*-HFP_22_) copolymer, but the carbon black or crosslinked moiety, because few F^−^ ions were formed.

**Figure 5:**
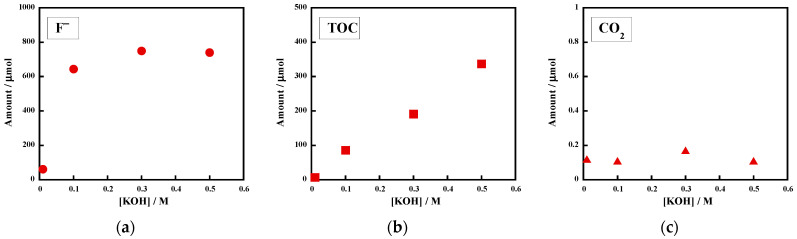
Effect of initial KOH concentration on the amounts of (**a**) F^−^ ions, (**b**) TOC, and (**c**) CO_2_. The crosslinked FKM (30.0–30.5 mg) was reacted with 0.01–0.50 M of KOH at 250 °C for 6 h. The reaction atmosphere was argon.

Figure 5 displays the KOH concentration dependences of (a) the F^−^ amount in the reaction solution, (b) the TOC amount in the reaction solution, and (c) the CO_2_ amount in the gas phase generated from the reactions of the crosslinked FKM at 250 °C for 6 h.

**Figure 6:**
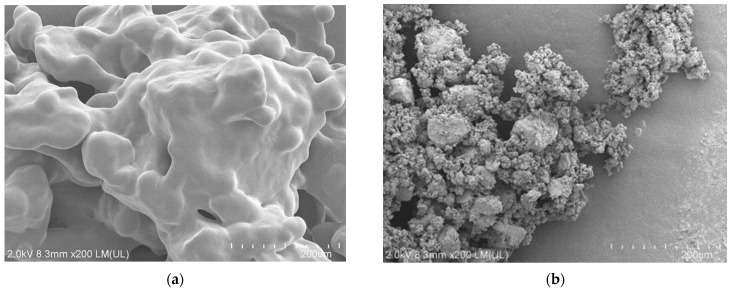
SEM images (200×) of (**a**) uncrosslinked FKM and (**b**) crosslinked FKM.

When 0.10 M of KOH was used, the amount of F^−^ ions was 644 μmol, or an 88% yield. This yield was higher than the corresponding value (68%) for the uncrosslinked FKM (Figure 1a), suggesting that the crosslinked moiety and/or carbon black of the crosslinked FKM enhanced the reactivity. A remarkable difference between the uncrosslinked and crosslinked FKMs was observed on their surfaces. Figure 6 shows the SEM images of these FKMs before the reactions.

Although these FKMs were powdered via freeze-crushing for the experiments, the particles tended to reaggregate. This phenomenon was more pronounced in the uncrosslinked FKM. The SEM images clearly show that the surface area of the crosslinked FKM (Figure 6b) is larger than that of the uncrosslinked FKM (Figure 6a). This is likely the reason why the crosslinked FKM was more reactive than the uncrosslinked FKM in superheated water.

The rise in the KOH concentration increased the level of F^−^ formation (Figure 5a). At 0.30 M of KOH, the amount of F^−^ ions reached 749 μmol, that is, a 102% yield. Therefore, the fluorine content of the crosslinked FKM (30.5 mg) was completely transformed into F^−^ ions in the aqueous phase. The TOC amount increased monotonically with the increasing KOH concentration (Figure 5b). At 0.50 M of KOH, the TOC amount was 337 μmol, or 26% of the carbon molar amount in the initial crosslinked FKM (Table 1, entry 9). A trace amount of CO_2_ was observed, and this amount was almost constant (0.1–0.2 μmol) for all the tested KOH concentrations, ranging from 0.01 to 0.50 M (Figure 5c).

**Figure 7:**
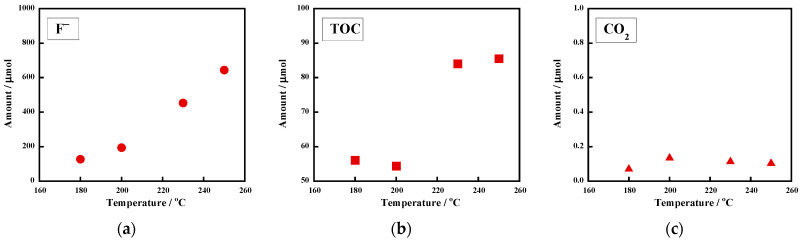
Temperature dependences of the amounts of (**a**) F^−^ ions, (**b**) TOC, and (**c**) CO_2_. The crosslinked FKM (30.0–30.5 mg) was heated with 0.10 M of KOH for 6 h. The reaction atmosphere was argon.

The temperature effect on the reactivity of the crosslinked FKM with 0.10 M of KOH was also examined (Figure 7). For a reaction at 180 °C, the F^−^ amount was 127 μmol, or an 18% yield. The rising temperature increased the degree of F^−^ formation (Figure 7a). At 250 °C, the amount reached 644 μmol, or an 88% yield. This yield was higher than the F^−^ yield (68%) obtained for the uncrosslinked FKM under the same conditions (Figure 2a). While the F^−^ amount monotonically increased from 180 °C to 250 °C (Figure 7a), the TOC amount dramatically increased above 230 °C (Figure 7b). This tendency was the same as that observed for the uncrosslinked FKM (Figure 2a,b), suggesting that defluorination occurred first, followed by polymer chain scission. Under these conditions, little CO_2_ was detected in the gas phase (Figure 7c). When the reaction time was extended to 18 h at 230 °C, the F^−^ amount reached 684 μmol, or a 93% yield. For a reaction at 250 °C for 18 h, the F^−^ amount reached 687 μmol (a 95% yield).

Therefore, the almost complete fluorine mineralization of the crosslinked FKM was achieved through the use of a rather low KOH concentration, specifically 0.10 M.

#### 2.2.2. Complete Fluorine Mineralization at 200 °C

To achieve complete fluorine mineralization at even lower temperatures with a relatively low KOH concentration, reactions using 0.20 M of KOH were performed. When the crosslinked FKM was reacted at 200 °C for 18 h, the amount of F^−^ ions was 732 μmol, which corresponds to a 100% yield (Table 1, entry 10, the average value of two reactions). Hence, even at such a low temperature (200 °C) and a low KOH concentration (0.20 M), the crosslinked FKM was successfully mineralized under the same conditions as those used for the uncrosslinked FKM (Table 1, entry 7). The crosslinked FKM contains carbon black and a crosslinked moiety. However, its presence did not inhibit fluorine mineralization. In their review paper [22], Schuster et al. concluded that elastomers, and therefore fluorinated elastomers, should be vulcanized with the goal of further facilitating devulcanization. However, the fluorine content in the crosslinked FKM used here can be completely mineralized through the use of superheated water.

### 2.3. Synthesis of CaF_2_

**Figure 8:**
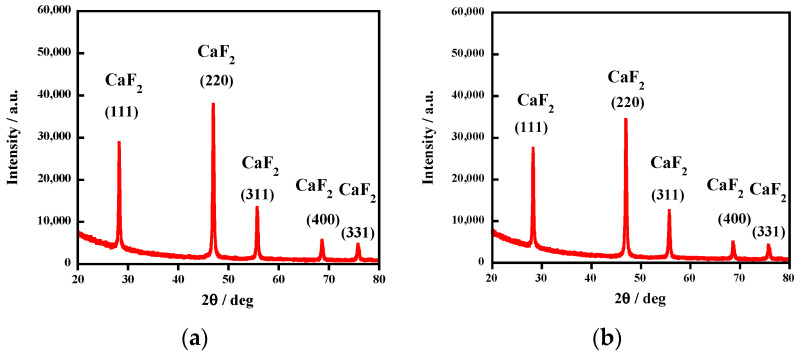
XRD patterns of CaF_2_ powders obtained from (**a**) uncrosslinked FKM and (**b**) crosslinked FKM.

We reacted the F^−^ ions released following complete mineralization with Ca(OH)_2_. After the uncrosslinked FKM was mixed with aqueous KOH (0.50 M) and heated at 250 °C for 6 h under argon, the liquid phase was collected through centrifugation. Next, we introduced Ca(OH)_2_, of which the molar amount was equal to that of the F^−^, into the liquid. The formed precipitate was purified using hydrochloric acid (1.0 M), rinsed in deionized (milli-Q) water, and dried using a rotary pump. The X-ray diffraction (XRD) pattern of the dried particles revealed only CaF_2_ peaks (Figure 8a). The mass of this powder demonstrated that all the fluorine atoms constituting the uncrosslinked FKM were recovered into CaF_2_. That is, a 100% CaF_2_ yield was achieved.

The same procedure was applied to the solution from the crosslinked FKM, reacted under the same conditions as those described above. The treatment also yielded X-ray spectroscopically pure CaF_2_ (Figure 8b) with a 93% yield.

## 3. Materials and Methods

### 3.1. Materials

All chemicals, unless otherwise noted, were purchased from Fujifilm Wako Pure Chemical (Osaka, Japan) and used as received. Argon (99.99%), O_2_ (99.999%), and a standard gas mixture (CO_2_ and N_2_) were supplied by Taiyo Nippon Sanso (Shinagawa, Japan). The uncrosslinked FKM was supplied by Daikin Industries (Osaka, Japan). Combustion ion chromatography, carried out by the vendor, revealed that the fluorine percentage in the FKM was 64.5 wt%, which was slightly lower than the ideal value (66.0 wt%). On the other hand, the carbon percentage of the FKM was 31.7 wt%, determined via elementary analysis (vendor information). This value was also slightly lower than the ideal value (32.1 wt%). The crosslinked FKM, consisting of a poly(VDF_78_-*co*-HFP_22_) copolymer (cured using peroxide) and carbon black, was also supplied by Daikin Industries. The weight ratio of the poly(VDF_78_-*co*-HFP_22_) copolymer to carbon black was 100/40, and the fluorine and carbon percentages were 45.6 wt% and 50.8 wt%, respectively, as determined using combustion ion chromatography and elementary analysis. The F^−^ and CO_2_ yields and the remaining TOC ratios of the reactions for these FKMs were calculated according to their corresponding analytical values.

### 3.2. Superheated Water Reactions

A high-pressure reactor (internal volume, 31 mL) was employed. A gold vessel was fitted to this reactor to avoid any contamination from the stainless steel (the reactor metal). Each FKM (29.8–30.5 mg) and an aqueous KOH (10 mL, 0.01–0.50 M) were added to the gold vessel. Then, argon gas was added (0.60 MPa), and the reactor was heated in an oven to the reaction temperature (180–250 °C). After a designated reaction time, the reactor was rapidly cooled to ca. 25–30 °C using a blower. The gas in the head space of the reactor was collected in a Tedlar^®^ bag and analyzed using gas chromatography/mass spectrometry (GC/MS). The resulting content in the vessel was separated through centrifugation. The collected solution was analyzed using ion chromatography. TOC measurements were also carried out. The separated residue was dried and characterized using Raman spectroscopy and carbon analysis. Reactions using dioxygen were also carried out as control experiments.

### 3.3. Analytical Methods

The amounts of F^−^ were quantified using ion chromatography. The details are described elsewhere [21]. The amounts of TOC were monitored using an N/C 3100 BU TOC analyzer (Analytik Jena, Jena, Germany). The gas phase was analyzed using a QP2010 SE GC/MS system (Shimazu, Kyoto, Japan) with an Rt-Q-BOND column (Restek, Bellefonte, PA, USA). Helium was used as a carrier gas. The gas sample was introduced into the system in the split mode (20/1 by volume ratio), and the detection was performed in the full scan mode (*m*/*z* range, from 2.0 to 200). The column temperature was changed according to the following program: maintenance at 30 °C (5 min), elevation to 200 °C at 20 °C min^−1^, and then maintenance at 200 °C (20 min). XRD patterns of the prepared CaF_2_ were measured using a MultiFlex diffractometer (Rigaku, Tokyo, Japan) with copper Kα radiation. The surface of the FKM particles before the reactions was monitored through scanning electron microscopy (SEM) using an SU-8020 instrument (Hitachi, Tokyo, Japan).

## 4. Conclusions

We investigated the mineralization of fluoroelastomers (FKMs) in superheated water using KOH. The fluorine content of both uncrosslinked and crosslinked FKMs was efficiently converted into F^−^ ions in the reaction solution through reactions using low KOH concentrations (0.10–0.50 M) at 200–250 °C. When the uncrosslinked or crosslinked FKMs were reacted at a rather low temperature (200 °C) with 0.20 M of KOH for 18 h, these FKMs were successfully mineralized, both showing F^−^ yields of 100%. Although the crosslinked FKM contained carbon black, the fluorine mineralization of this FKM was not inhibited. The addition of Ca(OH)_2_ to the reaction solutions after the superheated water treatment at 250 °C for 6 h with 0.50 M of KOH yielded pure CaF_2_, identified via XRD, with 100% and 93% yields from the uncrosslinked and crosslinked FKMs, respectively.

Today, superheated water technology is being implanted on the pilot or commercial scale for the recycling of waste plastics [31]. Simultaneously, the economic evaluation of this technology is now being undertaken [32,33]. Wang et al. highlighted that, in the recycling of nonmetallic components from e-waste, superheated water technology and gasification technology have a lower environmental impact than pyrolysis, but their energy consumption is higher [33]. A drawback of superheated water technology is its energy cost. In particular, the heating of the reactor from room temperature to the reaction temperature is an energy-intense stage. Therefore, although here we achieved the complete fluorine mineralization of the FKMs at 200 °C, further studies on life cycle assessment, including energy analysis, along with the scale-up of reactions, are being undertaken by our team.

## Figures and Tables

**Table 1:** Products from the reactions of FKMs and PVDF.

Entry	Polymer [Initial Amount/mg]	KOH Conc. /M	Atmosphere	*T*/ °C	*P*/ MPa	Time /h	F^−^/μmol [Yield/%]	TOC/μmol [Ratio/%]	CO_2_/μmol [Yield/%]
1	uncrosslinked FKM [29.8]	0	argon	250	4.4	6	0.9 [0]	<0.2 ^a^ [0]	1.6 [0]
2 ^b^	PVDF [30.0]	0	argon	250	4.4	6	1.4 [0]	3.7 [0]	2.1 [0]
3	uncrosslinked FKM [30.5]	0.10	argon	250	4.6	6	702 [68]	80 [10]	15.9 [2]
4 ^a^	PVDF [30.4]	0.125	argon	250	4.4	6	794 [83]	62 [7]	0.8 [0]
5	uncrosslinked FKM [30.5]	0.50	argon	250	4.5	6	1060 [102]	244 [31]	0.08 [0]
6	uncrosslinked FKM [30.1]	0.50	O_2_	250	4.3	6	1040 [102]	123 [15]	1.8 [0]
7	uncrosslinked FKM [29.9 ± 0.1]	0.20	argon	200	2.3	18	1008 ± 2 [100 ± 1]	138 ± 18 [17 ± 3]	0.2 [0]
8	crosslinked FKM [30.0]	0	argon	250	4.5	6	8.8 [1]	<0.2 ^a^ [0]	33.1 [3]
9	crosslinked FKM [30.5]	0.50	argon	250	4.4	6	740 [101]	337 [26]	0.1 [0]
10	crosslinked FKM [30.5]	0.20	argon	200	2.3	18	732 ± 3 [100]	258 ± 12 [20 ± 1]	0.1 [0]

^a^ Below detection limit. ^b^ Taken from [21].

## Data Availability

Not applicable.

## Sample Availability

Not available.
